# Supplementary material for: Dynamic Wavelength-Tunable Photodetector Using Subwavelength Graphene Field-Effect Transistors
Source: Sci Rep. 2017 Apr 4;7:45873. doi: 10.1038/srep45873 (PMC5379207; doi:10.1038/srep45873)
Supplement: Supporting Information [file srep45873-s1.pdf]

Dynamic Wavelength-Tunable Photodetector Using Subwavelength Graphene Field-Effect Transistors

François Léonard†, Catalin D. Spataru†, Michael Goldflam‡, David W. Peters‡, Thomas E. Beechem‡*

†Sandia National Laboratories, Livermore, CA, 94551, United States

‡Sandia National Laboratories, Albuquerque, NM, 87185, United States

*fleonar@sandia.gov

Supporting Information

1. Overall theoretical/computational approach

Figure S1 shows the overall approach for calculating the photocurrent. The first two isolated steps consist in calculating the dark band-bending and the light fields in the device geometry. The third step is a one-shot calculation of the photocurrent using input from the first two steps.

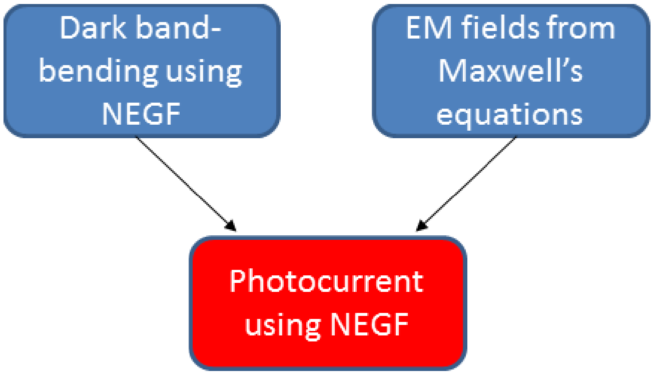

Figure S1. Illustration of the general approach to calculate the photocurrent.

2. Calculation of dark band-bending

Our approach to calculate the band-bending has been documented extensively in the case of carbon nanotubes[1]. We describe it here paying attention to the details specific to graphene. We calculate the dark band-bending by performing a self-consistent calculation between the electrostatic potential and the graphene charge. The electrostatic potential is obtained by solving Poisson's equation $\nabla\cdot(\varepsilon(x,y,z)V(x,y,z))=-\rho(x,y,z)$ with the source charge $\rho(x,y,z)$ being the graphene charge. The coordinate system is given in Figure 1 of the main text. The dielectric constant varies spatially in the y direction at the interface between the oxide and the vacuum above it. Fixed values of the potential are set at the source, gate, and drain electrodes. At the left, right, and top of the simulation cell, we set a boundary condition of zero derivative on the electrostatic potential. The vacuum space above the device is chosen large enough that the results are converged with respect to that height. Computationally, we use an in-house custom code based on a non-uniform grid to solve Poisson's equation. The calculation is accelerated by using a periodic unit cell in the z direction, and using a fast Fourier transform algorithm for that direction.

At the boundaries between the graphene and the contacts or the oxide, the graphene is vertically separated by a van der Waals gap of 0.3 nm on either side. We have discussed the modeling of contacts in this manner previously[2].

Once the electrostatic potential is obtained, we use the non-equilibrium Green's function approach to obtain the charge on the graphene. (Since we only need the zero bias band-bending, in practice the calculations are accelerated by only using equilibrium Green's functions.) This is done within a tight-binding model with nearest-neighbor interaction of value 2.5 eV. The graphene

atoms are located at vertical position $y = h$, and the electrostatic potential is evaluated at the location of the graphene atoms $V(x_i, h, z_i)$ where (x_i, h, z_i) is the location of atom *i*. We then add $-eV(x_i, h, z_i)$ as a diagonal term in the tight-binding Hamiltonian. The total charge on each graphene atom is obtained by solving the usual NEGF equations with semi-infinite graphene leads. In practice, the calculations are performed by considering a graphene nanoribbon of index (n,m) corresponding to the notation familiar from carbon nanotubes. In our case, the zigzag edge is parallel to the contact edges, and therefore the nanoribbon is of (n,0) type. We solve the NEGF equations independently for each band $p = 1,...,n$ and sum the total charge from each band. We found that $n = 100$ gives good convergence of the total charge.

Once we have the total charge on each atom, we use a 3D Gaussian profile at each atom site to distribute the charge and create the charge density $\rho(x, y, z)$ that enters Poisson's equation. The electrostatic potential is re-calculated, and used as the new input to the charge calculation. This process is repeated until self-consistency is achieved using the Broyden algorithm.

3. Calculation of electromagnetic fields

Using COMSOL Multiphysics, we obtain the electromagnetic fields by solving Maxwell's equations for a periodic array having the device geometry shown in Fig. 1a of the main text. The device is excited by a uniform plane wave polarized in the x direction. The source, drain, and gate contacts are modeled as perfect electric conductors and the fields at the device edges are forced to satisfy periodic boundary conditions. For the results in the main text, a non-dispersive model of a generic oxide is employed with ϵ_1=3.9 and ϵ_2=0. Using this simplified optical model yields more

generally applicable results, which may be extended to a range of more complex dielectric materials.

To examine the effects of using more realistic oxide properties, we ran identical simulations employing a fully dispersive SiO_2 model. The optical properties employed, as well as the resulting contact enhancement ratio, are shown in Figure S2. The enhancement ratio shows a strong dependence on the permittivity of SiO_2, suggesting the important role of the dielectric in the device optical response. However, the enhanced electric fields at the contacts is still a general feature across the wavelength range.

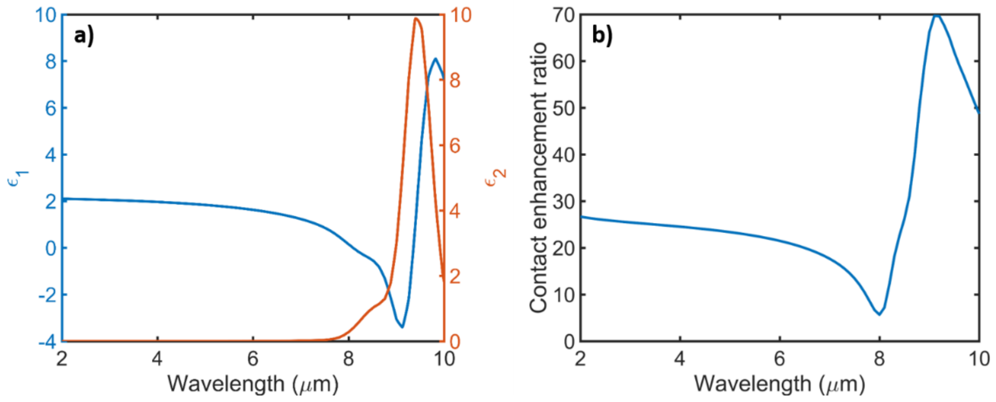

Figure S2. a) Real (ϵ_1) and imaginary (ϵ_2) SiO_2 permittivity employed in COMSOL modeling. b) Contact enhancement ratio obtained from simulations using realistic SiO_2 optical properties.

4. Calculation of photocurrent

We calculate the photocurrent using the NEGF approach we previously developed in the context of carbon nanotubes[3], but modified for the particular case of the graphene FET. For small light intensities, and in the absence of multi-photon processes, current in a device can be obtained from the expression

$$I = \frac{4e\gamma}{h}\int dE\,\mathrm{Re}\left\{G_{0,1}^{<(dark)}(E) + G_{N,N+1}^{<(dark)}(E)\right\} + \frac{4e\gamma}{h}\int dE\,\mathrm{Re}\left\{G_{0,1}^{<(ph)}(E) + G_{N,N+1}^{<(ph)}(E)\right\} \quad (1)$$

where $G_{i,j}^{<(dark)}$ is the Hamiltonian Green's function between tight-binding sites i and j in the absence of light, and $G_{i,j}^{<(ph)}$ comes from the linear expansion in the photon flux. The Green's functions in the dark correspond to those used when obtaining the self-consistent charge and potential in the device geometry, as discussed in section 2.

The photocurrent is defined as

$$I^{(ph)} = \frac{4e\gamma}{h}\int dE\,\mathrm{Re}\left\{G_{0,1}^{<(ph)}(E) + G_{N,N+1}^{<(ph)}(E)\right\} \quad (2)$$

with

$$G^{<(ph)} = G^{R0}\Sigma^{<(ph)}G^{R0\dagger} + G^{R0}\Sigma^{R(ph)}G^{R0}\Sigma^{<0}G^{R0\dagger} + G^{R0}\Sigma^{<0}\left(G^{R0}\Sigma^{R(ph)}G^{R0}\right)^{\dagger}. \quad (3)$$

In this equation $G^{R0} = \left[EI - H_0 - \Sigma^{R0} + i\eta\right]^{-1}$ with H_0 the bare Hamiltonian, Σ^{R0} the self-energy due to the semi-infinite graphene leads, and η a small positive energy. For the dark state we also have $\Sigma^{<0} = -2f\,\mathrm{Im}\Sigma^{R0}$ with f the Fermi distribution function.

The electron-photon interaction is captured by adding the $\vec{A}\cdot\vec{p}$ interaction to the Hamiltonian through the function[3]

$$\Sigma^{\{R,<\}(ph)}(E) = \alpha\sum_{pq} P_{lp}P_{qm}G_{pq}^{\{R,<\}0}(E - \hbar\omega) \quad (4)$$

with

$$P_{lm} = \delta_{l\pm1,m}\left[f^{\pm}(l) + f^{\pm}(m)\cos\left(\frac{\pi J}{M}\right)\right]. \quad (5)$$

In these equations, $f^{\pm}(l) = \mp 1 - \left(-1\right)^{l}$ and $\alpha = \dfrac{e^2 a^2 \gamma^2 F}{2\hbar\omega c\varepsilon}$ with $a = 0.071\,\text{nm}$ the smallest separation between adjacent lines of carbon atoms perpendicular to the transport direction, $\gamma = 2.5\ \text{eV}$ the tight-binding overlap integral, F the photon flux, $\hbar\omega$ the photon energy, c the speed of light, and ε the permittivity of free space.

Equation (5) is obtained by considering a graphene channel of finite width but with periodic boundary conditions in the direction perpendicular to the transport direction. In this case, there are *M* bands each described by a quantum number *J*. In practice, we use a (10000, 0) ribbon and we found that the photocurrent is converged by using ~500 bands.

In the case of the graphene FET device, the absence of an asymmetric built-in field implies that the photocurrent is zero unless a finite bias is applied. It is certainly possible to perform the NEGF calculations in the presence of a finite bias, but since the response is linear at small bias we can take advantage of the fast numerical calculations of the dark Green's functions and focus on the *photoconductance*, defined as

$$\sigma^{ph} = \left.\frac{\partial I^{(ph)}}{\partial V_{sd}}\right|_{V_{sd}=0} \tag{6}$$

We note that previous theoretical work[4] has demonstrated that such linear response functions in the presence of arbitrary interactions can be written as

$$\sigma^{ph} = \frac{4e^2\gamma}{hk_BT}\int dE T_{eff}\left(E\right) \tag{7}$$

where $T_{eff}\left(E\right)$ is an effective transmission function. In our case the effective transmission function can be written as

$$T_{eff}(E)=\left[\frac{f\left(E-\hbar\omega\right)-f\left(E\right)}{f\left(E-\hbar\omega\right)}\right]\Gamma\left(E\right)\mathrm{Im}\left[G^{R0}\left(E\right)\Sigma^{<(ph)}G^{R0\dagger}\left(E\right)\right]_{NN}\ . \tag{8}$$

This is the transmission function that we plot in the main text.

4. Responsivity

Traditionally the responsivity is defined as

$$\text{responsivity}=\frac{\text{photocurrent}}{\text{optical power}} \tag{9}$$

but since we calculate the photoconductance instead of the photocurrent, we define the photoconductance responsitivity as

$$\mathcal{R}=\frac{\sigma^{ph}}{P_{opt}} \tag{10}$$

where P_{opt} is the total optical power incident on the graphene channel.

5. Momentum conservation and vertical transitions

Here we explain how momentum conservation emerges from the above NEGF formalism when translational invariance is preserved. We use a single band, one-dimensional tight-binding chain since the Green's function is known analytically.

The Green's function for the chain is

$$G_{pq}^{R0}\left(E\right)=-\frac{i}{\hbar\nu}\mathrm{e}^{i\left(k_E|p-q|\delta\right)} \tag{11}$$

where p and q are the site indices and δ is the grid spacing. For a tight-binding interaction parameter γ the energy dispersion is given by

$$E=\frac{E_g}{2}+2\gamma\left[1-\cos\left(k_E\delta\right)\right] \tag{12}$$

with

$$\hbar\nu = 2\delta\gamma \sin\left(k_E\delta\right). \tag{13}$$

The expression for the self-energy due to the electron-photon interaction becomes (we only need the imaginary part to calculate the transmission)

$$\mathrm{Im}\Sigma_{lm}^{<ph} = -\frac{2\alpha}{\hbar\nu} f\left(E-\hbar\omega\right)\sum_{pq} P_{lp}P_{mk}\cos\left[k_{E-\hbar\omega}\left|p-q\right|\delta\right]. \tag{14}$$

For the tight-binding chain we have

$$P_{lm} = \begin{cases} +1 \text{ if } m = l+1 \\ -1 \text{ if } m = l-1 \\ \ 0 \text{ otherwise} \end{cases} \tag{15}$$

We can then obtain the transmission as

$$T_{eff}(E) \sim \frac{\sin\left(k_{E-\hbar\omega}\delta\right)}{\sin^2\left(k_E\delta\right)}\sum_{pq}\cos\left[k_{E-\hbar\omega}\left|p-q\right|\delta\right]\cos\left[k_E\left|p-q\right|\delta\right] \tag{16}$$

where we have omitted prefactors that do not influence our qualitative conclusions in order to more simply illustrate the main point.

Figure S3 shows a plot of Eq. (16) as a function of $\Delta k = k_E - k_{E-\hbar\omega}$ for a tight binding chain with $\gamma = 2.5$ eV, $\mathrm{E}_g = 1.5$ eV, and δ=0.1 nm. As can be seen from this plot, as the number of sites N increases, a sharper peak is observed at $\Delta k = 0$ signaling the enforcement of momentum conservation as the chain length becomes infinite.

The above derivation sheds light on the origin of the non-vertical transitions discussed in the manuscript. Indeed, for illumination of finite length, we expect non-vertical transitions as illustrated from the wings at non-zero Δk in Fig. S3. Furthermore, when the electrostatic potential is not uniform along the channel, the Hamiltonian Green's function will not have the simple form

in Eq. (11), also leading to non-vertical transitions. Figure 6 of the main text demonstrates these effects in detail for the actual case of the graphene FET.

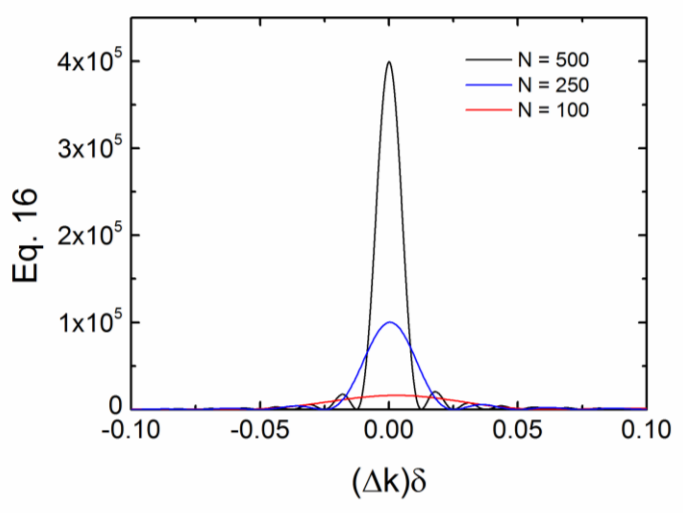

Figure S3. Equation 16 plotted for a tight binding chain with the number of sites *N* from 100 to 500. The peak at $\Delta k = 0$ is the signature of momentum conservation.

6. Graphene optical absorption

To test the validity of the NEGF formalism with the electron-photon interaction, we calculated the optical absorbance of graphene as a function of photon energy. In the NEGF formalism, the optical absorbance is given by

$$\Upsilon = \frac{1}{\pi\hbar}\frac{(ea_{cc}\gamma)^2}{16\hbar\omega c\varepsilon_0}\int dE\Sigma^{<(ph)}(E-\hbar\omega)G^{>}(E). \tag{17}$$

For this calculation, we considered a uniform electrostatic potential of -0.1 eV such that the Fermi level is 0.1 eV above the Dirac point, and used $\eta = 1\,\text{meV}$. Figure S4 shows the resulting optical absorbance, compared with the analytical expression[5]

$$\Upsilon = \frac{e^2}{4\hbar c\varepsilon_0}\frac{1}{1+(\hbar\omega/\Gamma)^2} + \frac{e^2}{8\hbar c\varepsilon_0}\left[\tanh\left(\frac{\hbar\omega+2E_F}{4k_BT}\right)+\tanh\left(\frac{\hbar\omega-2E_F}{4k_BT}\right)\right]. \tag{18}$$

The second term in this expression is due to interband transitions, which saturates to the well-known absorbance of ~2.3% at large photon energy. The first term is due to intraband transitions which are typically modeled with a phenomenological broadening energy Γ. Our numerical calculations using the NEGF approach give good agreement with this analytical expression. At large photon energy we approach the universal value of the absorbance of 2.3%, with the actual number being somewhat less due to the finite size of the system (200 nm illumination length in this case, which already requires significant computational resources to converge). At small photon energies, we have a Drude-like increase in absorbance that can be fitted to the first term of Eq. (18).

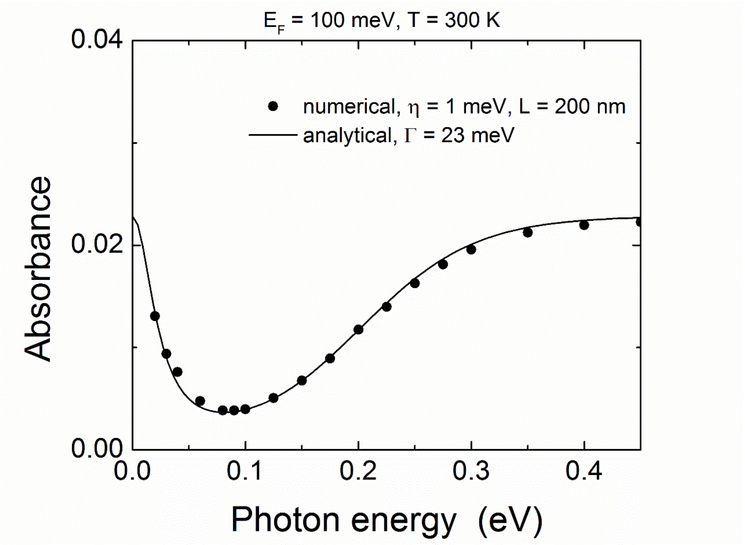

Figure S4. Optical absorbance of graphene calculated from the NEGF approach (solid circles) compared with Eq. (18).

(1) Léonard, F.; Stewart, D. A. *Nanotechnology* **2006,** *17*, 4699.
(2) Cummings, A. W.; Léonard, F. *ACS Nano* **2012,** *6*, 4494-4499.
(3) Stewart, D. A.; Léonard, F. *Phys. Rev. Lett.* **2004,** *93*, 107401.
(4) Datta, S. *Phys. Rev. B* **1992,** *46*, 9493-9500.
(5) Mak, K. F.; Ju, L.; Wang, F.; Heinz, T. F. *Solid State Commun.* **2012,** *152*, 1341-1349.
